# Genistein decreases cellular redox potential, partially suppresses cell growth in HL-60 leukemia cells and sensitizes cells to γ-radiation-induced cell death

**DOI:** 10.3892/mmr.2014.2611

**Published:** 2014-10-08

**Authors:** IN GYU KIM, JIN SIK KIM, JAE HA LEE, EUN WIE CHO

**Affiliations:** 1 Department of Radiation Biology, Environment Radiation Research Group, Korea Atomic Energy Research Institute, Yuseong, Daejeon 305-600, Republic of Korea; 2 Department of Radiation Biotechnology and Applied Radioisotope, University of Science and Technology, Yuseong-gu, Daejeon 305-353, Republic of Korea; 3 Bioconvergence Department, Korea Conformity Laboratories, Yoensu-gu, Incheon 406-840, Republic of Korea; 4 Biomedical Translational Research Center, Korea Research Institute of Bioscience and Biotechnology, Yuseong-gu, Daejeon 305-806, Republic of Korea

**Keywords:** cell cycle transition, cytoplasmic nicotinamide adenine dinucleotide phosphate-dependent isocitrate dehydrogenase, genistein, reactive oxygen species, redox potential, radiation

## Abstract

Various mechanisms have been proposed to underlie the cellular activity of genistein, based on biological experiments and epidemiological studies. The present study demonstrated that genistein inhibited the expression of cytoplasmic nicotinamide adenine dinucleotide phosphate (NADP)-dependent isocitrate dehydrogenase (*cICDH*), thus increasing levels of intracellular reactive oxygen species (ROS) in human promyeloid leukemia HL-60 cells. In genistein-treated cells, the cellular redox potential (GSH/GSSG) was significantly decreased. This decrease in redox potential was caused by significant downregulation of the *cICDH* gene, generating the reducing equivalents (NADPH) for maintenance of cellular redox potential and cellular ROS level, which may regulate cell growth and cell death. Genistein-induced ROS partially induced rapid transition into the G2/M phase by upregulation of p21^wap1/cip1^ and apoptotic cell death. Treatment of cells with N-acetylcysteine, a well-known antioxidant (ROS scavenger), not only partially restored cell growth and inhibited cell cycle arrest in G2/M, but also prevented apoptotic cell death. By contrast, normal lymphocytes did not significantly progress into the G2/M phase and radiation-induced cell death was inhibited by genistein treatment. Therefore, genistein and γ-irradiation together synergistically cause cell death in leukemia cells, however, genistein has a radioprotective effect in normal human lymphocytes. In conclusion, it was suggested that genistein selectively functions, not as an antioxidant, but as a pro-oxidant in HL-60 cells. This property can increase ionizing radiation-induced cell cycle arrest and sensitivity to apoptotic cell death in human promyeloid leukemia HL-60 cells, but does not cause significant damage to normal cells.

## Introduction

Genistein (4′,5,7-trihydroxyisoflavone), a naturally occurring soybean isoflavone glycoside with a heterocyclic diphenolic structure similar to estrogen (1), is considered to be a potent anticancer agent (2,3). The potential importance of genistein was highlighted by a previous study that reported an increased consumption of soy in Asia resulting in increased levels of isoflavone in serum, which is closely associated with a reduced risk of prostate cancer (4). Genistein has been demonstrated to inhibit growth of tumor cell lines derived from various malignancies, including breast cancer, prostate cancer, head and neck squamous cell carcinoma, melanoma and leukemia (5–11). Genistein is considered to affect diverse cell functions, for example, it has been demonstrated to trigger cell cycle arrest and apoptotic cell death through inactivation of NF-κB and activation of caspase-3 in prostate cancer cells, as well as to have potent anti-angiogenic activity, inhibiting tumor cell proliferation (12–14). Previous studies have also suggested that genistein suppresses tumor cell growth through the inhibition of tyrosine protein kinases (15), topoisomerases I and II (16,17) and the expression of mRNAs of cell cycle-related genes (18) in different cell types. By contrast, under other circumstances, apoptotic cell death was inhibited in the presence of genistein. Several previous studies revealed that genistein was able to prevent apoptotic cell death via its antioxidant properties (19,20). Genistein inhibited UV irradiation-induced oxidative stresses and neuronal damage resulting from production of reactive oxygen species (ROS). It also inhibited methylglyoxal-induced apoptotic cell death in a human mononuclear cell model, and inhibited methylglyoxal-induced DNA damage and ROS production *in vitro*. Animal experiments further confirmed the protective effect of genistein on methylglyoxal-induced cell injury (21).

Intracellular ROS, including superoxide, hydrogen peroxide and hydroxyl radicals, are generated following exposure to ionizing radiation, selected chemotherapeutic agents (Taxol and etoposide), hyperthermia, inhibition of antioxidant enzymes (including thioredoxins, catalase, superoxide dismutases and glutathione-linked peroxidase) and depletion of cellular reductants, including nicotinamide adenine dinucleotide phosphate (NADPH), reducing equivalents and glutathione (GSH) (22–25). Therefore, ROS are involved in numerous biological and pathophysiological situations, including aging and inflammation. ROS have high chemical reactivity and, thus, damage lipids, proteins, as well as mitochondrial and nuclear DNA, which can lead to cell cycle arrest (26,27). Furthermore, ROS generation can induce apoptotic cell death through depletion of intracellular reduced GSH and protein thiols, and loss of mitochondrial membrane potential (25,27). The present study used human promyeloid leukemia HL-60 cells to examine the intracellular signal mechanisms involved in genistein-induced cell growth arrest and cell death.

## Materials and methods

### Cell culture and growth

Genistein and *N*-acetylcysteine were purchased from Sigma (St. Louis, MO, USA). A stock solution of genistein was prepared in dimethyl sulfoxide. Stock solution of N-acetylcysteine was prepared in phosphate-buffered saline (PBS). Working solutions were prepared by dilution of stock solutions in culture medium. HL-60 human promyeloid leukemia cells (4×10^4^/ml) were grown as suspension cultures in RPMI-1640 medium (Gibco, Scotland, UK) supplemented with 10% fetal bovine serum (FBS; Hyclone, Logan, UT, USA) and 100 U/ml penicillin/streptomycin (Sigma) in a humidified atmosphere containing 5% CO_2_ at 37°C for 24 h. Normal human lymphocytes were isolated from peripheral blood of healthy human males. Blood was added to Ficoll-Paque (Amersham Pharmacia Biotech, Uppsala, Sweden) and centrifuged at 400 × g for 20 min. The lymphocyte layer was collected using a micropipette and diluted with serum-free RPMI-1640[0]. The diluted cell suspension was centrifuged at 70 × g for 10 min. Lymphocytes (4×10^4^/ml) were cultured in RPMI-1640 supplemented with 10% FBS, 100 U/ml penicillin/streptomycin and 5 μg/ml phytohemagglutinin (Sigma) in a humidified atmosphere containing 5% CO_2_ at 37°C for 24 h. Cell growth and viability were determined using a trypan blue (Sigma) exclusion test.

### γ-irradiation

Cells (2×10^5^/ml) pretreated with 20 μM genistein for 6 h and untreated cells were irradiated with a single dose of 2 Gy (dose rate, 0.2 Gy/min) or 5 Gy (dose rate, 0.5 Gy/min) and then cultured in a humidified atmosphere containing 5% CO_2_ at 37°C.

### Measurement of intracellular ROS level

HL-60 cells (4×10^4^ cells/4 ml) were cultured in T25 flasks and treated with 20 μM genistein for 0, 12, 24 and 48 h. Harvested cells (5×10^5^) were treated with 10 μM chloromethyl-2′,7′-dichlorofluorescein diacetate (DCFH-DA) for 30 min in the dark and then washed with PBS. The intracellular ROS level was measured using the FACScan (Beckman-Coulter Instruments Inc., Brea, CA, USA) and visualized using a fluorescence microscope (Leica, Heidelberg, Germany).

### Measurement of intracellular GSH level

In order to determine the total intracellular levels of reduced (GSH) and oxidized (GSSG) forms of GCH, a GSH assay kit (Cayman Chemical, Ann Arbor, MI, USA) was used. HL-60 cells (1×10^7^) were used for each experiment. Concentrations of GSH and GSSG were calculated from the typical standard curves. The detectable range was 0.2–6.0 nmol/ml.

### Reverse transcription-polymerase chain reaction (RT-PCR) analysis

TRIzol^®^ reagent (Invitrogen Life Technologies, Grand Island, NY, USA) was used to isolate total RNA from 5×10^6^ HL-60 cells according to the manufacturer’s instructions. Total RNA (1 mg) was added to a 20-μl reaction mixture containing Maxime RT PreMix (iNtRON Biotechnology, Seongnam, Korea) and 10 pmole primers. Primers used were *cICDH*, forward 5′-TTGGATCCAAAATGTCCAAAAAA-3′ and reverse 5′-ATGAATTCAAGTAGTCAGAACGT-3′; β-actin, forward 5′-CA TCCTCACCCT GAAGTACCC-3′ and reverse 5′-AGCCTGGATAGCAACGTACATG-3′. RT-PCR was performed in a thermal cycler (Apollo, San Diego, CA, USA) under conditions of 45°C for 30 min, followed by 94°C for 5 min, and 25 cycles of 94°C for 1 min, 52°C for 1 min and 72°C for 1 min. PCR products were separated on a 1.5% agarose gel and visualized with ethidium bromide staining.

### Propidium iodide staining for analysis of apoptotic cell death and cell cycle status

HL-60 cells (2×10^5^) were suspended in 2 ml ice-cold 50% ethanol and maintained at 4°C for 40 min. Fixed cells were harvested by centrifugation, at 1,000 × g for 10 min, and resuspended in 800 μl PBS. Subsequently, 100 μl RNase (1 mg/ml) and 100 μl propidium iodide (400 μg/ml) were added to the cell suspension, and cells were incubated at 37°C for 30 min. This allowed for the discrimination of live cells from apoptotic and necrotic cells. Analysis of apoptotic cell death was performed using a FACScan (Beckman-Coulter Instruments Inc.) equipped with a single 488-nm argon laser (Beckman-Coulter Instruments Inc.). The percentages of apoptotic cells and the cell cycle distribution were calculated using MultiCycle for Windows software (Phoenix Flow Systems, San Diego, CA, USA).

### Western blot analysis

The protein extract sample was separated in a 12.5% denaturing polyacrylamide gel, followed by transfer onto nitrocellulose membranes (GE Healthcare Bio-Sciences, Pittsburgh, PA, USA). Membranes were incubated with monoclonal anti-human p21^wap1/cip1^, polyclonal anti-human Bcl-2-associated X protein (Bax), polyclonal anti-human β-actin (Cell Signaling Technology, Inc., Danvers, MA, USA) and polyclonal anti-human B-cell lymphoma 2 (Bcl-2; Santa Cruz Biotechnology, Inc., Dallas, TX, USA) at room temperature for 2 h, and then with secondary antibodies (anti-mouse or anti-rabbit immunoglobulin G horseradish peroxidase-conjugated; Cell Signalling Technology, Inc.) at room temperature for 1 h. Membranes were washed four times with Tris-buffered saline with Tween 20 and protein bands were visualized using an ECL detection kit (Amersham Pharmacia Biotech). Protein concentrations were determined by the Lowry method.

### Morphological analysis

Human blood lymphocytes and HL-60 cells were treated with genistein and γ-radiation, followed by culture for 48 h. Cytochalasin-B (4 μg/ml, Sigma) was added 20 h after γ-irradiation. Cells were harvested and resuspended in hypotonic 0.075 M KCl for 3 min. Cells were centrifuged again and Carnoy’s fixative (American MasterTech, Lodi, CA, USA) was gently added. Cells were then mounted on clean slides and air-dried. The slides were stained with Giemsa (Sigma) solution and observed under a light microscope (Leica).

## Results

### Effect of genistein on HL-60 cell proliferation and intracellular ROS generation

**Figure 1:**
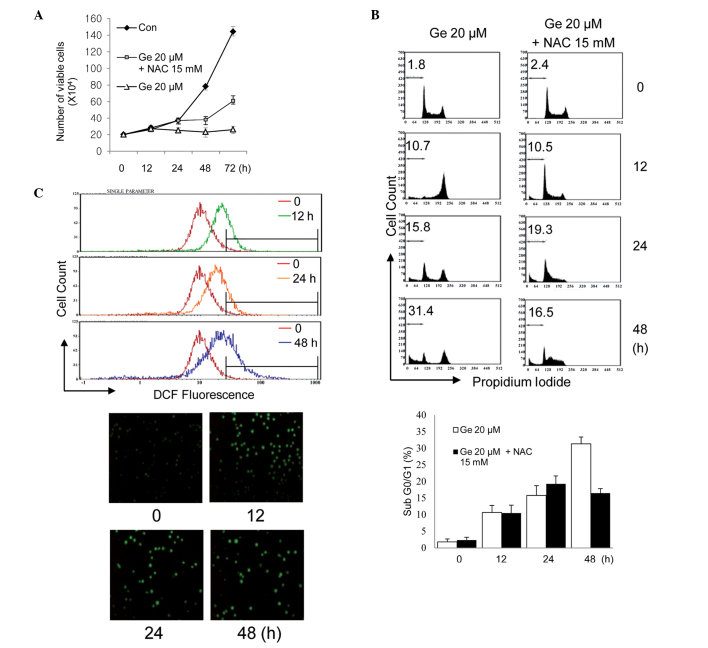
Effect of genistein on the proliferation of HL-60 cells and intracellular ROS generation. (A) Growth curve for human promyeloid leukemia HL-60 cells exposed to genistein in the presence or absence of *N*-acetylcysteine (15 μM). Values are presented as the mean ± SD of three independent experiments. (B) Effect of genistein (20 μM) on the level of apoptotic cell death in HL-60 cells. Values are expressed as the mean ± SD of three independent experiments. (C) Effect of genistein (20 μM) on intracellular ROS generation in HL-60 cells. DCFH-DA fluorescence was determined by flow cytometry and visualized using a fluorescence microscope 12, 24 and 48 h after the treatment. ROS, reactive oxygen species; SD, standard deviation; DCFH-DA, chloromethyl-2′,7′-dichlorofluorescein diacetate.

Fig. 1A shows the time-dependent response of HL-60 cells to exposure to 20 μM genistein in the presence or absence of 15 mm N-acetylcysteine, a sulfur-containing antioxidant compound. Genistein-treated cells demonstrated significant retardation of cell growth and, based on time-lapse images, apoptotic cell death was significantly increased (Fig. 1B). A strictly regulated cellular level of ROS is essential for the proliferation of tumor cell growth, therefore an imbalance of ROS affects cell growth arrest and cell death (28). The level of cellular ROS in HL-60 cells was examined to determine whether genistein causes a change in cellular ROS level and is associated with cell growth inhibition in these cells. The oxidant-sensitive probe DCFH-DA was used, which permits detection of various oxygen-derived free radicals by flow cytometry and fluorescence microscopy (Leica). As shown in Fig. 1C, intracellular ROS were significantly elevated in HL-60 cells following treatment with 20 μM genistein for 12, 24 and 48 h, compared with the control HL-60 cells. However, cells partly recovered from genistein-induced cell growth inhibition and death when exposed to genistein in the presence of N-acetylcysteine, a cell-permeable ROS scavenger. This finding indicates that genistein led to upregulation of cellular ROS in HL-60 cells and that this affected cell growth.

### Expression of cICDH

**Figure 2:**
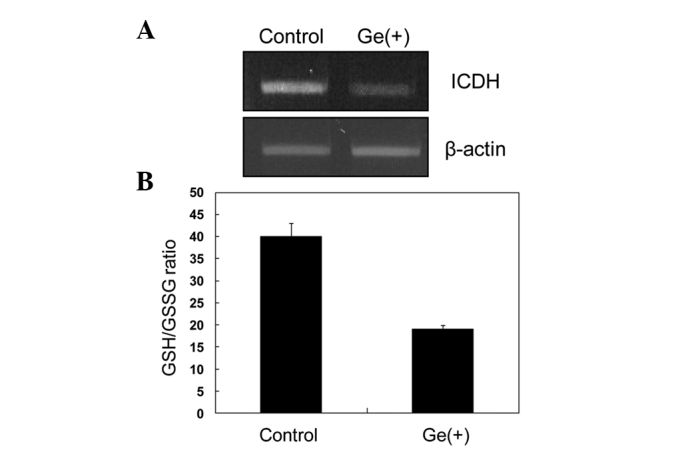
Effect of Ge(+) on the expression of the reducing-equivalent-generating cytoplasmic nicotinamide adenine dinucleotide phosphate-dependent *cICDH* in HL-60 cells. (A) Reverse transcription polymerase chain reaction was used to analyze the gene expression of *cICDH* in HL-60 cells. The housekeeping gene β-actin was used as an internal control. (B) Intracellular GSH/GSSG ratio was determined in genistein-treated HL-60 cells. Values are presented as the mean ± standard deviation of three independent experiments. Ge, genistein; ICDH, isocitrate dehydrogenase.

The present study investigated how genistein induced elevation of the intracellular ROS level. In cells, the cellular redox potential (GSH/GSSG ratio) is an important factor in the homeostatic regulation of intracellular ROS, which, in turn, is important in cell signaling for proliferation. In addition, redox potential is also a critical factor in the control of cell growth in various cancer cell lines. Reducing equivalents (NADPH) generated by *cICDH* or glucose-6-phosphate dehydrogenase are indispensable for the regeneration of oxidized GSH, kithioredoxin and other molecules of this type. Therefore, to ascertain the role of genistein in the generation of ROS, intracellular redox potential, as well as *cICDH* involved in the regulation of cellular redox status was examined. Genistein treatment decreased the transcriptional levels of *cICDH* and, thus, significantly decreased the GSH/GSSG ratio (Fig. 2A and B). The level of *cICDH* gene expression in the genistein-treated HL-60 cells was only 20% that of the control cells and, consequently, resulted in a decrement by half in the GSH/GSSG ratio.

### Pro-oxidant activity of genistein results in G2/M phase arrest and apoptosis

**Figure 3:**
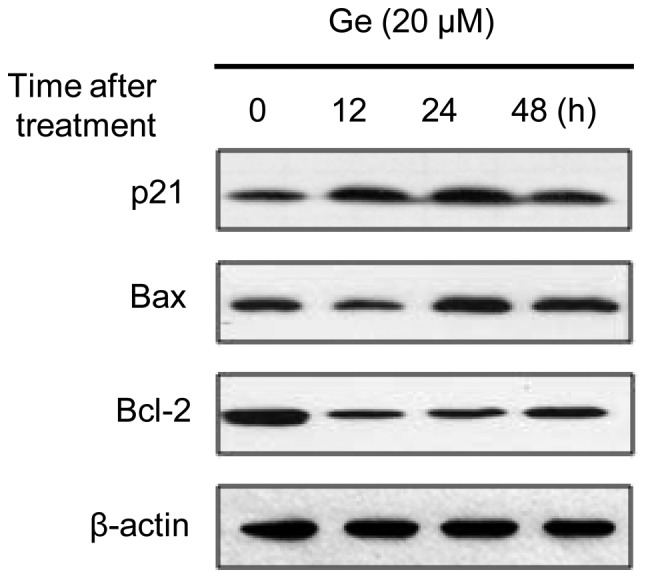
Effect of genistein on the cellular levels of the apoptosis-related proteins p21^waf1/cip1^, Bax and Bcl-2. Ge, genistein; Bcl-2, B-cell lymphoma 2; Bax, Bcl-2 associated × protein.

Genistein was suggested to induce cell cycle arrest in the G2/M phase, which leads to inhibition of cell growth (29). To investigate whether ROS are involved in genistein-induced G2/M phase transition and cell death in the HL-60 cell line, cell cycle progression was analyzed. HL-60 cells were treated for 48 h with 20 μM genistein. Following 12 h of genistein treatment, cell cycle progression into the G2/M phase was most prominent. In total, 63% of HL-60 cells treated with genistein were in the G2/M phase, with a concomitant decrease in cells in the G0/G1 phase from 32 to 1%. An increase in the sub-G0/G1 peak (hypodiploid apoptotic cells) was also noted. Cell death exponentially increased 48 h after genistein treatment. By contrast, addition of N-acetylcysteine inhibited or delayed genistein-induced G2/M phase progression and prevented apoptotic cell death. *N*-acetylcysteine also significantly induced S phase arrest, enabling repair of genistein-induced damage (Table I). These data indicated that genistein-induced G2/M phase arrest is caused by elevated intracellular ROS. Based on these findings, the levels of expression of p21^WAF1/Cip1^ and cyclin B1, two molecules involved in cell cycle progression, were evaluated by western blot analysis. As shown in Fig. 3, genistein increased the level of p21^WAF1^/^Cip1^ after 12, 24 and 48 h of treatment, resulting in a 2–3-fold increase in expression. The effect of genistein on the Bcl-2 family of proteins, which are associated with apoptotic cell death, in HL-60 cells was also examined. Upregulation of the proapoptotic protein Bax in genistein-treated cells and downregulation of the antiapoptotic protein Bcl-2 was observed.

### Effects of γ-irradiation on human promyeloid leukemia HL-60 cells and normal human lymphocytes

**Figure 4:**
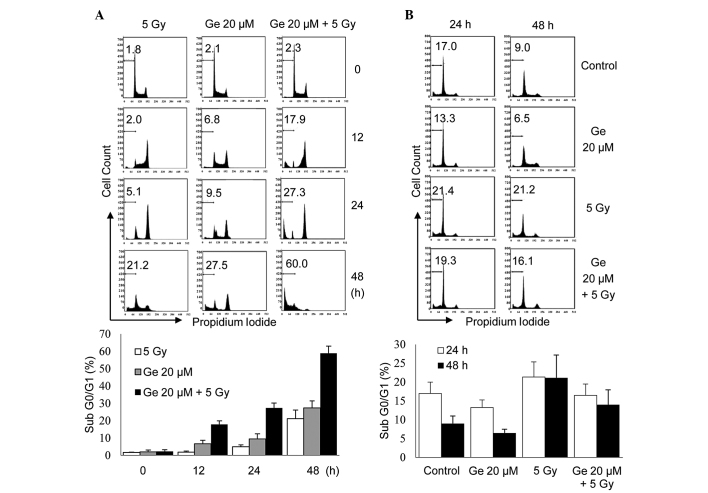
Ge and γ-radiation-induced apoptotic cell death in human promyeloid leukemia HL-60 cells and normal human lymphocytes. The change in hypodiploid content produced by Ge treatment (20 μM) and γ-irradiation (5 Gy) in (A) HL-60 cells and (B) normal lymphocytes. Values are expressed as the mean ± standard deviation of three independent experiments. Ge, genistein.

As shown in Fig. 4, the effect of sensitization to γ-radiation in apoptotic cell death was investigated in genistein-treated HL-60 cells by measuring the change in hypodiploid content. The effect of genistein on radiation-induced damage in normal lymphocytes was also investigated. Following γ-irradiation at a dose of 5 Gy (dose rate, 0.5 Gy/min), HL-60 cells progressed into the G2/M phase and arrested there. After 48 h, cells either undergo cell death or are repaired and re-enter the G1 phase; at that time point, ~21% of cells underwent cell death. Genistein-treated HL-60 cells also progressed into the G2/M phase and, 48 h after genistein treatment, cell death was observed in 27% of cells. When administered together, genistein and γ-radiation synergistically increased cell death to a higher level than either agent alone (Fig. 4A). Notably, no such synergistic effect was observed in normal human lymphocytes. Compared with its radiosensitizing effect on HL-60 leukemia cells, genistein had a radioprotective effect on normal lymphocytes after 24 and 48 h of treatment (Fig. 4B).

### Differences in morphology of human promyeloid leukemia HL-60 cells and normal human lymphocytes

**Figure 5:**
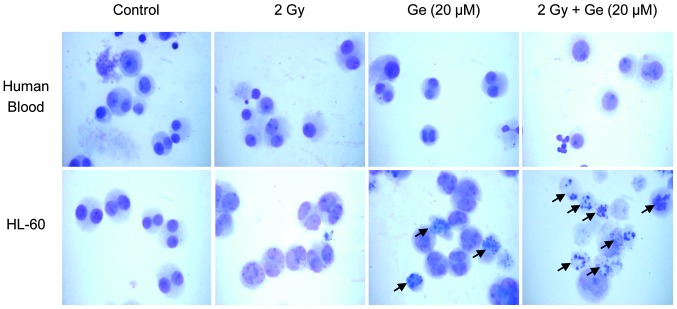
Ge and γ-radiation-induced morphological alterations between human promyeloid leukemia HL-60 cells and normal human lymphocytes. Arrows indicate separated apoptotic cells (apoptotic bodies) Ge, Genistein.

Finally, it was confirmed that genistein had different effects on radiation-induced damage in promyeloid leukemia HL-60 cells and normal human lymphocytes by the detection of apoptotic bodies. At a dose of 2 Gy, a negligible number of γ-radiation-induced apoptotic bodies were detected in normal lymphocytes. However, radiation treatment partially induced initiation of apoptosis in HL-60 cells. Genistein clearly induced the formation of apoptotic bodies in certain HL-60 cells. However, it did not affect apoptotic body formation in normal lymphocytes. Genistein (20 μM) and γ-radiation synergistically increased apoptotic body formation in HL-60 cells. Furthermore, this combination treatment resulted in the formation of apoptotic bodies in HL-60 cells. However, significant numbers of apoptotic bodies were not observed in normal lymphocytes under any condition (Fig. 5).

## Discussion

Genistein is known to induce differentiation, cell cycle arrest, apoptosis and inhibition of tumor cell growth, and also possesses anti-angiogenesis and antioxidant activities (9–11). Cell cycle arrest is a characteristic of eukaryotic cells and the cell cycle progresses through different phases commonly referred to as checkpoints (12). Cell cycle checkpoints are transient delays in G_1_/S or G_2_/M transition that constitute a response to DNA damage by cellular stressors, including ROS, and allow time for the activation of repair mechanisms (13). Following repair of damaged DNA, cells resume cell cycle progression. However, if the damage is too severe, the cells may undergo apoptosis or irreversible senescence (14). Similarly to genistein, ROS cause DNA damage and induce G2/M phase arrest and apoptosis (22,25). The present study investigated whether ROS are involved in genistein-induced cell cycle arrest and cell death in the HL-60 cell line. To date, it has been hypothesized that genistein eliminates oxygen free radicals generated by toxic agents and hydrogen peroxide (28–31). In addition, it is well known that genistein inhibits topoisomerase II activity, which leads to its cleavage and thus induced G2/M phase arrest and apoptosis (30–32). However, additional mechanisms underlying the antioxidant activity and induction of apoptotic cell death by genistein remain to be elucidated. The present study concluded that, in human promyeloid leukemia HL-60 cells, genistein affects the cellular redox potential level, which is known to be important in the regulation of cellular physiology, including cell growth and differentiation.

The result from the present study that G_2_/M arrest in response to genistein treatment sensitizes HL-60 cells to γ-radiation-induced cell death, corroborates previous studies in DU145 human prostate cancer cells (33) and cervical cancer cells (34). Cells in the G_2_/M phase have been demonstrated to be more radiosensitive than cells in other phases of the cell cycle (35–37). Pretreatment with genistein arrests cells in G_2_/M, and thus, may increase their radiosensitivity, resulting in increased cell death, in addition to the direct cytotoxic effects of genistein and γ-radiation. The present study further addresses the role of the cellular redox potential and reducing equivalents-generating enzyme, *cICDH*, in the mechanism by which genistein enhances intracellular ROS and radiation-induced cell death. Intracellular GSH depletion or low GSH/GSSG ratio caused excessive intracellular ROS accumulation. Alternatively, the downregulation of the enzymes involved in GSH synthesis, and maintenance of the reduced GSH level may also result in ROS accumulation and, thus, sensitivity to γ-radiation and anticancer drugs. In the present study, total GSH increased with genistein treatment (data not shown). Furthermore, the level of the antioxidant enzyme thioredoxin also increased (data not shown). Despite elevated levels of these factors, genistein increased sensitivity to γ-radiation-induced cell death. These findings suggest that cellular redox potential (GSH/GSSG) may be a critical factor in this process. Although levels of GSH and thioredoxin increased, if the oxidized form is converted to the reduced form, cellular redox potential is not maintained at a steady state. NADP^+^-dependent *ICDH* is necessary for the maintenance of the cellular redox potential level at a steady state by production of the reducing equivalents (NADPH) (38). Therefore, the present study examined the expression of the *ICDH* gene by RT-PCR and confirmed that the expression level was significantly lower in genistein-treated cells compared with the controls.

It has been reported that genistein treatment combined with radiation enhances radiosensitivity in numerous cancer cell lines (37,38). In the present study, it was demonstrated that genistein also has a synergistic effect with γ-radiation on apoptosis in HL-60 cells. By contrast, genistein has a protective effect on normal lymphocytes. Cells respond to DNA-damaging agents by activating cell-cycle checkpoints, and cells in the G_2_/M phase of the cell cycle have been demonstrated to be more radiosensitive than cells in other phases (33–35). Several types of cancer cells are hypersensitive to γ-radiation in the G2/M phase, compared with normal cells, as they are deficient in DNA repair capacity (39–41). However, in normal human lymphocytes, neither genistein nor radiation alone promoted a decrease in the percentage of cells in G_0_/G_1_ and a concomitant increase in the percentage of cells in G_2_/M. This indicated that DNA damage by genistein or radiation is not critical in normal lymphocytes and, thus, cell cycle transition and arrest for repair is not required. This may explain why genistein did not have a synergistic effect on radiation-induced cell death. By contrast, genistein had a radioprotective effect in normal human lymphocytes as G2/M phase arrest did not occur. In conclusion, the results from the present study suggest that genistein does not act as an antioxidant, but as a pro-oxidant, in human promyeloid leukemia HL-60 cells. The pro-oxidant activity of genistein caused a rapid transition of HL-60 cells into the G2/M phase and, thus, inhibited cell proliferation and apoptotic cell death. In addition, the combination of genistein treatment and γ-irradiation demonstrated a synergistic effect on cell death in HL-60 cells, whereas genistein exhibited a radioprotective effect in normal lymphocytes.

## Figures and Tables

**Table I:** Cell cycle distribution of HL-60 cells following treatment with genistein and *N*-acetylcysteine.

Time following treatment (h)	Percentage of cells in			

	Sub-G0/G1	G0/G1	S	G2/M
Genistein^a^				
0	1.8	32.6	49.7	15.9
12	10.7	1.4	24.6	63.3
24	15.8	30.6	23.9	29.7
48	31.4	19.5	11.1	38.0
Genistein + *N*-acetylcysteine^b^				
0	2.4	34.9	47.0	15.7
12	10.5	41.2	41.0	7.3
24	19.3	32.1	46.2	2.4
48	16.5	12.6	66.8	4.1

a Genistein, 20 μM;

b *N*-acetylcysteine, 15 mm.
